# The validity and reliability of the sixth-year internal medical examination administered at the King Abdulaziz University Medical College

**DOI:** 10.1186/s12909-015-0295-4

**Published:** 2015-02-01

**Authors:** Hind I Fallatah, Ara Tekian, Yoon Soo Park, Lana Al Shawa

**Affiliations:** 1 Faculty of Medicine, King Abdulaziz University, Jeddah, Saudi Arabia; 2 Department of Medical Education, College of Medicine, University of Illinois, Chicago, IL USA

**Keywords:** Validity, Assessment, Undergraduate medical education

## Abstract

**Background:**

Exams are essential components of medical students’ knowledge and skill assessment during their clinical years of study. The paper provides a retrospective analysis of validity evidence for the internal medicine component of the written and clinical exams administered in 2012 and 2013 at King Abdulaziz University’s Faculty of Medicine.

**Methods:**

Students’ scores for the clinical and written exams were obtained. Four faculty members (two senior members and two junior members) were asked to rate the exam questions, including MCQs and OSCEs, for evidence of content validity using a rating scale of 1–5 for each item.

Cronbach’s alpha was used to measure the internal consistency reliability. Correlations were used to examine the associations between different forms of assessment and groups of students.

**Results:**

A total of 824 students completed the internal medicine course and took the exam. The numbers of rated questions were 320 and 46 for the MCQ and OSCE, respectively. Significant correlations were found between the MCQ section, the OSCE section, and the continuous assessment marks, which include 20 long-case presentations during the course; participation in daily rounds, clinical sessions and tutorials; the performance of simple procedures, such as IV cannulation and ABG extraction; and the student log book.

Although the OSCE exam was reliable for the two groups that had taken the final clinical OSCE, the clinical long- and short-case exams were not reliable across the two groups that had taken the oral clinical exams. The correlation analysis showed a significant linear association between the raters with respect to evidence of content validity for both the MCQ and OSCE, r = .219 P < .001 and r = .678 P < .001, respectively, and r = .241 P < .001 and r = .368 P = .023 for the internal structure validity, respectively. Reliability measured using Cronbach’s alpha was greater for assessments administered in 2013.

**Conclusion:**

The pattern of relationships between the MCQ and OSCE scores provides evidence of the validity of these measures for use in the evaluation of knowledge and clinical skills in internal medicine. The OSCE exam is more reliable than the short- and long-case clinical exams and requires less effort on the part of examiners and patients.

## Background

Exams are essential for assessing undergraduate medical students in their clinical years of study because their future careers as clinicians are dependent on their competency and knowledge [1-3]. However, the accuracy of the exams administered in undergraduate medical schools to assess students’ requisite knowledge and skills has been frequently questioned [2,3]. The ability of medical exams to assess each student with accuracy and fairness across students is also debatable [2,3]. Examination methods have evolved over the last two decades, and different exam and assessment methods are being developed to evaluate clinical-year medical students [2-6]. The final graduation requirements for clinical medical students typically involve the completion of a comprehensive exam that includes written and clinical components [3,7]. The written component of the exam may include multiple-choice questions (MCQ) and short-answer essay questions [1,3], whereas the clinical portion may involve a long- or short-case examination, the completion of an objective structured clinical examination (OSCE) or other modified forms of long-case examinations [2,3,5,7]. Prior studies have evaluated the degree to which the instrument or exam measures what it is supposed to measure (validity) and the reproducibility or consistency in the scores (reliability) of different examination methods used in clinical-year medical exams [3,4,7]. Moreover, previous reports have shown the correlation between the OSCE and the MCQ exams to be higher than the correlation of the MCQ and other forms of performance evaluations because the MCQ intends to measure cognitive knowledge, whereas the OSCE intends to measure the applicability of knowledge in performance [7-9]. The King Abdulaziz University (KAU) Faculty of Medicine is the second oldest medical college in Saudi Arabia. KAU is a leader and the mother college of another 13 medical colleges in the country [10]. The number of students enrolled in the KAU Faculty of Medicine is rapidly increasing, posing new challenges for student assessment to faculty members and college administrators. In addition, the faculty has been required to implement improvement plans to meet local and international medical school accreditation standards. These conditions have resulted in the implementation of major changes to the medical school’s curriculum. These changes included shifting the system of teaching in the Faculty of Medicine from a year-based system to an integrated block-based system. This transition involved modifying examination and assessment methods and administering OSCEs to clinical-year medical students enrolled in the four major clinical departments. The internal medicine rotation comprises two courses. One course is taken during the fourth year, and the second involves a full-semester rotation during the final, sixth year of study. The sixth-year internal medicine course is an 18-week course that includes clinic-based learning, case-based discussions on real patients, lectures, topic-based tutorials and an externship rotation with bedside discussion. Sixth-year medical student assessments consist of a summative mark that includes continuous assessments, mid-rotation exams, and final exams. The mid-rotation exams and final exams include both written and clinical components to ensure a thorough assessment of students’ knowledge and skills. The validity and reliability of clerkship examinations are essential to ensure the proper measurement of necessary competence and skills in a consistent manner.

The aim of this study is to evaluate the reliability and to present validity evidence for both the written and clinical exams administered to sixth-year medical students during the internal medicine rotation at the KAU Faculty of Medicine in 2012 and 2013. The scores for the MCQs and OSCE were obtained and correlated with the results of the summative exam. Messick’s unified validity framework was used to examine validity evidence, focusing on the content (difficulty, clarity in terms of language and item complexity), internal structure (internal-consistency reliability and relationship within components of the assessment), and relationships to other variables.

## Methods

This study was approved by the institutional review board of the KAU Faculty of Medicine and the Department of Internal Medicine to review the questions and student results.

### Curriculum structure

The study conducted a retrospective cohort analysis of sixth-year medical students’ internal medicine examinations administered in 2012 and 2013. The new teaching curriculum at KAU was established in 2007, and the first class of students to complete this curriculum graduated in 2012. In 2012 and 2013, four groups of students completed the final course on internal medicine and took both the midterm exams and final exams. In 2012, both the midterm exams and the final exams consisted of a written component that included MCQ sections and data OSCE stations with clinical pictures, ECGs, radiological illustrations and laboratory data. In addition, clinical skills were assessed via short and long cases in both the midterm and the final exams. In 2013, the MCQ and the OSCE data were similar to the data in 2012, but the final clinical exam was changed to one long-case exam for the midterm and clinical OSCE stations on simulated patients for the final exam. The MCQs used in both 2012 and 2013 were obtained from the department’s item bank. This item bank was revised and updated to be aligned with the new curriculum. Hence, recall questions, negative statement questions and questions with “all of the following” statements were omitted from the question bank and revised by the faculty’s quality department. Each OSCE station was designed by a faculty member who was an expert in the relevant discipline and subsequently reviewed by 11 members of the exam committee (including six senior and five junior instructors) to ensure adherence to teaching objectives, examine the blueprint, assess the content difficulty and confirm item clarity for both students and raters. Before the OSCE exam was administered, clear instructions and training were provided to all OSCE exam raters by the exam organizing team to ensure accurate and consistent scoring for all OSCE stations.

### Data collection

In 2012 and in 2013, four groups of sixth-year medical students (two groups each year) completed the new internal medicine curriculum and took the midterm exam and final exams. For each group of students, exam marks for each section and the overall scores were obtained (30 points for the midterm exam, 20 points for the continuous assessment, 20 points for the final MCQ exam and 30 points for the final clinical exam OSC or long and short cases). The exam questions were taken from the midterm and final exams for all four groups. (The written MCQ section and OSCE stations were evaluated by four raters who were faculty members of the department). To prevent possible scaling bias, the first group of raters included two senior examiners with more than 10 years of teaching and exam facilitation experience who were also members of the exam committee. One of the examiners had both a local and a UK post-graduate education background, and the other had a North American post-graduate education background. The second group of raters included two junior instructors with two to three years of teaching and exam facilitation experience; they were not members of the exam committee and had not been involved in exam question preparation or revision. One of these individuals had a local postgraduate training background, and the other had a North American post-graduate training background. The evaluators were instructed to rate each exam item in terms of its content.

To determine content validity, the raters were asked to score each item in relation to the course objectives and the blueprint of the exam. Items were rated for difficulty, clarity of language, appropriate length of the question, item complexity and the relationship of the item to other items that constituted the exam. Each type of content validity rating was given a score between 1 and 5. (A score of “1” denoted the lowest level of content validity evidence, and a “5” represented the highest.) Each rater completed the evaluation independently.

### Analysis

SPSS version 22 was used to analyze the data. The mean scores provided by the raters for each type of validity were obtained for both the MCQ and OSCE exams. A correlation analysis was conducted on the ratings provided by the two groups of evaluators for the MCQ and the OSCE sections separately and for the MCQ and OSCE together. The correlation analysis was also used to identify the relationship between the MCQ and OSCE sections and between both of these sections and the grades from the continuous assessment. The continuous assessment grades were correlated to the summative final results. We used t-tests to compare the differences in grades between the final clinical exam of long and short cases in 2012 and the final OSCE exam in 2013. For both, the total mark was 30% of the final summative result. A correlation analysis was used to obtain the association for both the MCQ and clinical exams among the different groups. Cronbach’s alpha was used to evaluate the internal-consistency reliability of the summative results for the years 2012 and 2013 separately, the assessment, the MCQ and the clinical exams.

## Results

### Descriptive statistics

A total of 824 students in 2012 and 2013, divided into four groups of sixth-year students (two male groups and two female groups), completed the internal medicine rotation.

The number of students in each group is shown in Table 1. The breakdown of exam questions used for the groups was as follows: 320 MCQ questions, 32 OSCE questions and, for the 2013 groups only, 14 clinical OSCE questions. In 2012, 888 short cases were provided in the midterm exam, and a similar number was used in the final exam along with 444 long cases. In 2013, a total of 402 long-case questions were given for the midterm exam.

**Table 1:** **Student performance by curriculum year for the final internal medicine exams**

Curriculum year	Total Number of students 846	Mean and SD for MCQ (final) Out of 20	Mean and SD for OSCE 2013 (final) AND FINAL CLINICAL (final) 2012 out 30	Mean and SD for continuous assessment out of 20	*Mean and SD for total final mark out of 100 (all year)
2013	402 (47.5%)	14.4 SD 2.68	22.34 SD 4.62	18.07 SD 2.56	77.23 SD 11.136
1st and 2nd semester	200 (23.6%) 202 (23.9%)				
2012	444 (52.5%)	14.0510 SD 2.61	21.08 SD 3.62	19.25 SD 1.56	74.40 SD9.98
1st and 2nd semester	230 (27.2%) 214 (25.3%)				
Standardized mean differences		0.34	1,3	−1.2	.28
P value		.28	.001	.001	.002

*The final summative mark is 100 (30 for the midterm exam, 50 for the final exam and 20 for the continuous assessment). The midterm exams were not compared between the students of 2012 and those of 2013, because approximately 24 students from the 2011 group had failed the course before the new curriculum was adopted; thus, these students took only the final exam with the new curriculum group of 2012.

Table 1 provides an overview of the students’ performance and the student sample size. Only the final exam scores were referenced because this allows for more accurate results, for two reasons. First, approximately 24 students from the 2011 group had failed the course before the new curriculum was adopted and thus took the final exam with the new curriculum group of 2012. Because the 2011 group did not take the midterm exam but had instead taken the continuous assessment and then the final exam, the grade for this group was calculated differently. The second reason is that some students from previous groups had missed either the final or midterm exam due to illness or other extreme circumstances and, with permission from the Vice Dean Committee, were allowed to take the final exam with the following group of students without repeating a full rotation. A significant difference in the final clinical exam scores was found between the two groups of students taking the short- and long-case final exams in 2012 (means of 20.48 and 22.20; SDs of 3.8 and 3.0, respectively; *P <* .001). In terms of content, the final OSCE scores between the two groups who had taken the final clinical OSCE exam in 2013 were not significantly different (means of 21.9 and 20.80; SDs 4.4 and 4.4, respectively; *P =* .83).

The validity evidence for the MCQ and the OSCE assessments is presented below, organized into (1) relationships to other variables, (2) content, and (3) internal structure.

### Relationships to other variables

The relationships between the MCQ and OSCE sections and between each of these sections and the summative results are shown in Table 2. Both the long- and short-case MCQ clinical exams and the OSCE exams significantly correlated with the final exam results (*r =* 0.824 and *r* = 0.93, respectively; *P <* .001 for both correlations).

**Table 2:** **Correlations for the relationships between assessments**

	Final clinical (2012) or OSCE (2013)	MCQ	Assessment
MCQ (final)	r 0.56 P < .001	-	r 0.34 P < .001
Assessment	r 0 .34 p < .001	r 0.34 p < .001	.
Final clinical (2012) or OSCE (2013)	-	r 0.56 p < .001	r 0 .34 p < .001
Summative final exam result	r 0 .93 P < 001	r 0.82 P <001	r 0.06P < .001

In 2013, a strong correlation was found between the clinical midterm long-case examination scores and the final clinical OSCE exam scores (*r =*0.66 and P < .001).

### Content evidence

The mean content validity ratings for the four raters together were 4.6 (SD = 0.4) for the MCQ component (320 items) and 4.5 (SD = 1.4) for the OSCE component (32 questions). The mean internal structure validity ratings, in support of content validity, from the four raters were 4.2 (SD 0.6) and 4.8 (SD 0.4) for the MCQ and OSCE sections, respectively. No differences were found between the senior expert examiners and junior examiners with respect to content and internal validity scores for the entire exam, including the MCQ and OSCE sections. This result is evident in the positive correlation found between the two groups of examiners, as shown in Table 3.

**Table 3:** **Differences between the two groups of evaluators**

	Pearson correlation and P value MCQ	Pearson correlation and P value OSCE	Pearson correlation and P value for MCQ and OSCE together
Content validity	r .219 P < .001	r .678 P < .001	r .276 P < .001
Internal structure validity	r .241 P < .001	r .368 P = .023	r .240 P < .001

The correlation analysis between the two senior examiners for all of the exam questions was positive (*r =* 0.14 and *P =* .01). A similar positive correlation was found between the two junior examiners (*r =* 0.14 and *P =* .014).

### Internal structure evidence

Internal-consistency reliabilities for the total assessment scores were calculated. Cronbach’s alpha for the four components of the total assessment score (midterm result, continuous assessment, MCQ, and clinical exam) on both long and short cases (2012) or OSCE (2013) was 0.63 and 0. 83 for 2012 and 2013, respectively; Cronbach’s alpha for the long and short case clinical exam was 0.44 and 0.71, respectively, for the OSCE.

Using Pearson correlations, there was significant association in the MCQ exam results between the students from 2012 and 2013, *r* = 0.44; *p <* .001. There was a modest correlation between the scores obtained on the OSCE (*r* = 0.16; *p =* 0.03). However, no statistically significant correlation was obtained between the two groups of students who had clinical long- and short-case exams in 2012 (*r* = −0.06; *p* = 0.36).

## Discussion

This study shows that replacing the traditional long- and short-case clinical examinations with the OSCE increased the reliability and validity of the KAU internal medicine clinical exam.

Validity evidence is organized and presented using Messick’s unified validity framework and focusing on relationships to other variables, content, and internal structure. In this regard, the study shows that the current MCQ internal medicine exams for sixth-year students at KAU show evidence of strong validity. These findings reflect recent tendencies toward the adoption of standardized exams and validated questions [3,7,11,12]. Complementing the findings of Auewarakul et al. [7], our data from the KAU Faculty of Medicine show a significant correlation between the exams’ written and the clinical sections and between these exam components and the continuous assessment.

This finding may reflect individual student performance because student marks for different parts of the exam tended to correlate with the continuous assessment scores.

The MCQ examination results were not significantly different between the 2012 and 2013 student groups. This finding was further supported by the correlation analysis between the students across the two years. However, a significant difference was found between the 2012 and 2013 clinical exam results. (The short- and long-case format was used in 2012, and the OSCE format was employed in 2013). Furthermore, examining the differences between the two student groups for each year, the clinical short- and long-case exam scores differed between the two students groups in 2012, but the OSCE exam results did not differ between the two student groups in 2013. This finding was also supported by the poor correlation among the 2012 students and the significant positive Pearson correlation among the 2013 students who took the OSCE exam. Similar previous studies have shown that long-case clinical exams are not reliable and that OSCE exams are more valid and reliable [2,3]. However, OSCEs are sometimes thought to be unreliable due to rater differences and potentially poor associations between the OSCE stations [3,11,12].

Cronbach’s alpha, which reflects the internal consistency of scores, was higher in 2013 than in 2012, which shows that the OSCE is more reliable than the traditional clinical exams in terms of enhancing the internal consistency reliability of the summative student assessment results.

Multiple authors have shown that the MCQ and OSCE exams are valid methods for assessing medical students in different years of study [5,6,11,12]. The validity evidence calculations provided in this report demonstrate that both the MCQ and OSCE exam scores were significantly correlated across the two groups of examiners with respect to content validity. Furthermore, the fact that the content validity calculation used in this report takes into account the internal medicine course exam blueprint adds more supporting evidence for the exam’s validity, which has been shown in previous studies [5,7].

As shown by the number of long and short cases employed in 2012, examining a large student body necessitates the recruitment of a large number of real patients and significant efforts on the part of examiners and patients. This issue was improved significantly in 2013 with the introduction of simulated patients to clinical OSCE stations.

### Limitations and areas for improvement

One limitation of this study relates to the inclusion of continuous assessment scores in the final total score because the accuracy of the continuous assessment score is difficult to judge when evaluating a large group of students, which is the current situation at the KAU Faculty of Medicine. A plan to overcome this challenge has been developed by the internal medicine department, and this method will be in effect in the instruction of the 2014/2015 student group. The new format will involve small-group teaching modules that include only five to six students per session. As part of the continuous assessment, each student will be given the opportunity to perform at least 6–8 one-on-one long-case presentations to a faculty member who will provide feedback. Moreover, additional analyses of validity evidence, including the factorial structure of the assessments, will be supplemented as areas for future study. Identifying potential differences between the groups studied (the types of OSCEs used in the assessment) is another area that will need to be examined over time. Another plan for curricular improvement that is currently in preparation to continue improving the educational system is the allocation of a mentor to every fourth-year student, because the fourth year of study constitutes a student’s first clinical year until graduation at the end of the sixth year. These changes are expected to positively affect students’ performance in the exams, particularly the clinical components.

## Conclusion

MCQ and OSCE exams have greater validity evidence, which supports the use of their scores for the assessment of undergraduate medical student performance over traditional clinical exams. OSCE exams are usually effective for smaller numbers of students, but our study showed that OSCE can also be effective for large numbers of students if it is well planned and appropriately implemented. Our findings may be valuable to KAU in implementing the standardization and validation of exams in all clinical departments. These findings are also relevant to other national and regional faculties of medicine that have a similarly large number of students every year, which makes exam organization and standardization a potential challenge.
